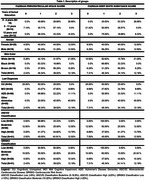# Educational Levels and Cardiovascular Risk Scores to predict Deep White Matter Hyperintensities in Fazekas Scale

**DOI:** 10.1002/alz70856_107398

**Published:** 2026-01-09

**Authors:** Thaise Vallesca Queiroz, Giovanna Correia Pereira Moro, Caio Mendes Ribeiro, João Marcos Silva Borges, João Henrique Fonseca, Aline Siqueira de Souza, Gabriela Tomé Oliveira Engelmann, Joice Coutinho de Alvarenga, Filipe Campos e Souza, Marco Aurélio Romano‐Silva, Jonas Jardim de Paula, Maria Aparecida Camargos Bicalho, Bernardo de Mattos Viana

**Affiliations:** ^1^ Federal University of Minas Gerais, Belo Horizonte, Minas Gerais, Brazil; ^2^ Cog‐Aging Research Group, Universidade Federal de Minas Gerais (UFMG), Belo Horizonte, Minas Gerais, Brazil; ^3^ Undergraduate Medicine, Faculty of Medicine, Universidade Federal de Minas Gerais (UFMG), Belo Horizonte, Minas Gerais, Brazil; ^4^ Older Adult's Psychiatry and Psychology Extension Program (PROEPSI), School of Medicine, Universidade Federal de Minas Gerais (UFMG), Belo Horizonte, Minas Gerais, Brazil; ^5^ Undergraduate medicine, Faculty of Medicine, Universidade Federal de Minas Gerais (UFMG), Belo Horizonte, Minas Gerais, Brazil; ^6^ Neurotec R National Institute of Science and Technology (INCT‐Neurotec R), Faculty of Medicine, Universidade Federal de Minas Gerais (UFMG), Belo Horizonte, Minas Gerais, Brazil; ^7^ Cog‐Aging Research Group, 31, Minas Gerais, Brazil; ^8^ Sciences Applied to Adult Health Postgraduate Program, School of Medicine, Universidade Federal de Minas Gerais (UFMG), Belo Horizonte, Minas Gerais, Brazil; ^9^ Molecular Medicine Postgraduate Program, School of Medicine, Universidade Federal de Minas Gerais (UFMG), Belo Horizonte, Minas Gerais, Brazil; ^10^ Older Adult's Psychiatry and Psychology Extension Program (PROEPSI), School of Medicine, Universidade Federal de Minas Gerais (UFMG), Belo Horizonte, Minas Gerais, Brazil, Belo Horizonte, MG, Minas Gerais, Brazil; ^11^ Neurotec R National Institute of Science and Technology (INCT‐Neurotec R), Faculty of Medicine, Universidade Federal de Minas Gerais (UFMG), Belo Horizonte, Minas Gerais, Brazil; ^12^ Department of Psychiatry, School of Medicine, Federal University of Minas Gerais, Belo Horizonte, Minas Gerais, Brazil; ^13^ Department of Internal Medicine, School of Medicine, Federal University of Minas gerais, Belo Horizonte, Minas Gerais, Brazil; ^14^ Department of Clinical Medicine, Faculty of Medicine, Universidade Federal de Minas Gerais (UFMG), Belo Horizonte, Minas Gerais, Brazil; ^15^ Geriatrics and Gerontology Center Clinical Hospital of Universidade Federal de Minas Gerais, Belo Horizonte, Minas Gerais, Brazil; ^16^ Older Adult's Psychiatry and Psychology Extension Program Federal University of Minas Gerais, Belo Horizonte, Minas Gerais, Brazil; ^17^ Cog‐Aging Research Group, Belo Horizonte, Minas Gerais, Brazil

## Abstract

**Background:**

Cardiovascular risk factors (CVR) are associated with an increased risk in developing dementia through lifespan. Additionally, magnetic resonance imaging (MRI) can assess the cerebrovascular burden and atrophy patterns that are essential for dementia's differential diagnosis.

**Objective:**

To conduct an analysis of the relationship of sociodemographic factors, white matter burdens with FAZEKAS visual scale on brain MRI, and CVR estimates.

**Methods:**

This is a cross‐sectional study, using data from the Cog‐Aging cohort study from 2018 to 2024. Forty‐one participants were recruited. MRI was analysed by two experienced and independent physicians to determine Fazekas deep white matter (DWM) and periventricular white matter (PVWM) T2 hyperintensities scores. The Cardiovascular Risk Score (QRISK3) and the Atherosclerotic Cardiovascular Disease (ASCVD) were selected to estimate CVR. Differences in Fazekas scores between CVR, diagnosis, and sociodemographic groups were explored with Mann‐Whitney and Kruskal‐Wallis analysis. Significant associations were further explored in Logistic Regression adjusted for education to determine the probability of the calculator results predicting changes in the FAZEKAS. Skin color, age and gender were removed from the regression because they are variables used in both calculators to assess risk. This study was approved by the UFMG Ethics Committee.

**Results:**

The mean age was 76.65 years (SD= 7.36) and the median years of education was 7 (IQR= 8.00). Differences in Fazekas scores were observed only for DWM between educational level groups. Logistic regression of CVR, showed that the QRisk (OR=1.067, *p* = .027) was slightly better at predicting DWM score of 2 or more, than ASCVD (OR=1.053, *p* = .023). When adjusted for education, both CVR scores lost significance, but the QRisk model was slightly better (AUC= .809, Nagelkerke R2= .361 / vs .787 and .329). Logistic Regression model with only educational level was significant (OR= .810 p.= .008) and when adjusted with CRV scores, it maintained significance (OR= .823, *p* = .017 QRisk; OR= .844, *p* = .036 ASCVD).

**Conclusion:**

These findings suggest that the educational level variable was better to predict a worse DWM (≥2) score than CVR scores. These are preliminary results and more studies with a higher sample with cardiovascular risk scores and MRI are needed.